# Supplementary material for: Formation of three-dimensional bicontinuous structures via molten salt dealloying studied in real-time by in situ synchrotron X-ray nano-tomography
Source: Nat Commun. 2021 Jun 9;12:3441. doi: 10.1038/s41467-021-23598-8 (PMC8190292; doi:10.1038/s41467-021-23598-8)
Supplement: Supplementary file 1 — Supplementary Information [file 41467_2021_23598_MOESM1_ESM.pdf]

**Supplementary Information:**

# Formation of Three-dimensional Bicontinuous Structures via Molten Salt Dealloying Studied in Real-Time by *In Situ* Synchrotron X-ray Nano-Tomography

Xiaoyang Liu[1,#], Arthur Ronne[1,#,*], Lin-Chieh Yu[1,2], Yang Liu[1,2], Mingyuan Ge[3], Cheng-Hung Lin[1], Bobby Layne[4], Phillip Halstenberg[5,6], Dmitry S. Maltsev[5], Alexander S. Ivanov[6], Stephen Antonelli[3], Sheng Dai[5,6], Wah-Keat Lee[3], Shannon M. Mahurin[6], Anatoly I. Frenkel[1,4], James F. Wishart[4], Xianghui Xiao[3], Yu-chen Karen Chen-Wiegart[1,3,*]

1. Department of Materials Science and Chemical Engineering, Stony Brook University, Stony Brook, NY, USA

2. Department of Chemistry, Stony Brook University, Stony Brook, NY, USA

3. National Synchrotron Light Source II (NSLS-II), Brookhaven National Laboratory, Upton, NY, USA

4. Chemistry Division, Brookhaven National Laboratory, Upton, NY, USA

5. Department of Chemistry, University of Tennessee, Knoxville, TN, USA

6. Chemical Sciences Division, Oak Ridge National Laboratory, Oak Ridge, TN, USA

#These authors contributed equally to this work.

*Corresponding authors: Karen.Chen-Wiegart@stonybrook.edu, arthur.ronne@stonybrook.edu

## X-ray nano-tomography visualization of dealloying and coarsening in molten salt

The 2D and 3D views of the sample showing the evolution of pores' and metals' morphologies while being heated in molten salts. The dealloying and coarsening processes led to a formation of a bicontinuous structure with an open porous structure.

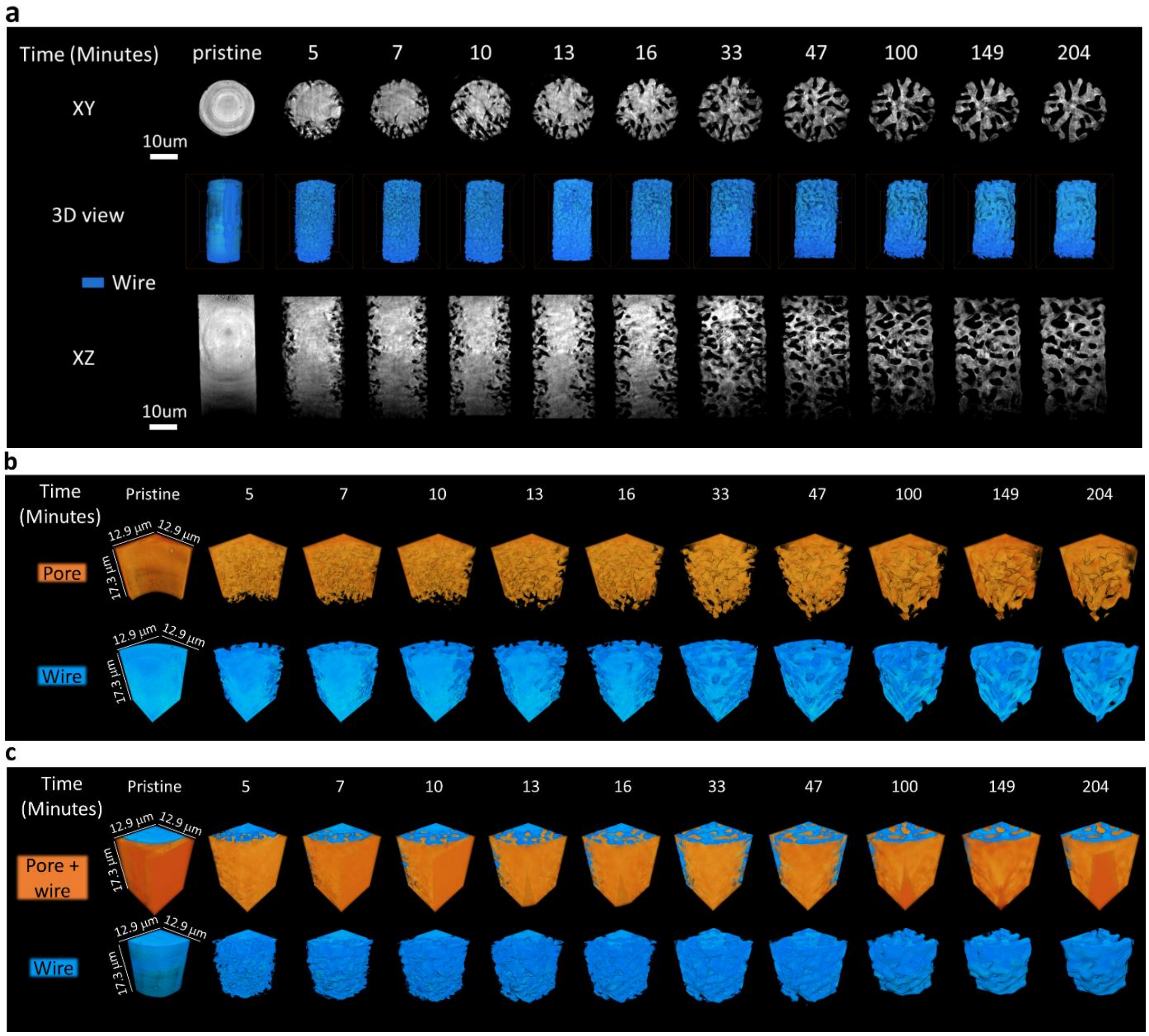


**Supplementary Figure 1: a** The wire sample *in situ* heating morphology change in pseudo cross-sectional views (XY and XZ planes) and 3D view. **b** Volume of interest (viewing from the center of the wire outwards): The pore and metal morphologies changed as a function of the reaction time. Pores gradually propagated from the wire-and-molten-salt interface to the center of the wire. At the later stage, the sizes of the pores and ligaments gradually increased due to the coarsening effect. **c** Volume-of-interest view (viewing from the surface of the wire inwards): The *in situ* visualization clearly shows the ligaments' morphological evolution.

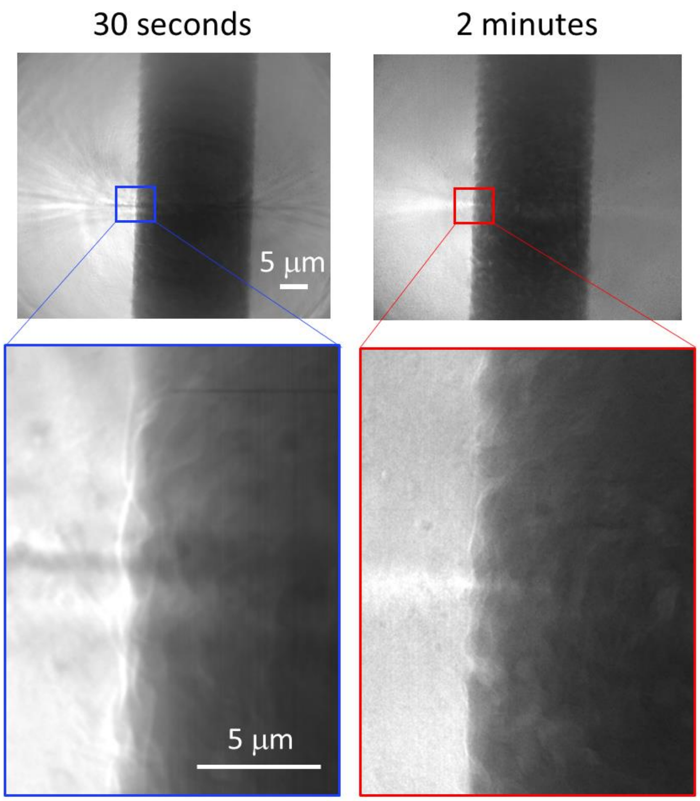


**Supplementary Figure 2:** The 2D projection views with enlarged local view at 30 seconds and 2 minutes show that the dealloying process was relatively rapid.

**Supplementary Table 1:** Dealloying distance *vs*. dealloying time quantification

| Time ($t$, minutes) | Dealloying Distance ($l$, μm) | Standard deviation for dealloying distance (μm) | Dealloying distance$^2$ ($l^2$, μm$^2$) | Standard deviation for dealloying distance$^2$ (μm$^2$) |
|---|---|---|---|---|
| 5.12 | 3.74 | 0.71 | 13.98 | 0.51 |
| 7.57 | 5.24 | 0.59 | 27.44 | 0.35 |
| 10.02 | 6.02 | 0.61 | 36.21 | 0.38 |
| 13.38 | 7.21 | 0.89 | 52.00 | 0.79 |
| 16.58 | 8.18 | 0.85 | 66.84 | 0.73 |
| 22.02 | 9.27 | 0.56 | 85.85 | 0.32 |
| 26.73 | 10.12 | 0.81 | 102.41 | 0.66 |

For step (iv), the long-range diffusion of the Cr ions produced by dealloying at the alloy-salt interface will also alter the dealloying rate. The long-range diffusion rate influences the local activity (effective concentration) of the Cr ions at the metal-salt interface. Note that the potential for the anodic reaction ($E_a$), $M \rightarrow M^{n+} + ne^-$, according to Nernst equation is:

$$E_a = E_a^0 - \frac{RT}{nF} ln \frac{a_{M^{n+}}}{a_M}, \qquad (1)$$

where $E_a^0$ is the corresponding standard electrode potential, $R$ is the gas constant, and $a_{M^{n+}}$ and $a_M$ are the activity for Cr ions and Cr in the alloy, respectively. When the dealloying electrochemical (anodic) reaction occurs, if Cr ions accumulate locally ($a_{M^{n+}}$ increased) due to a relatively slow diffusion rate, it would cause an increase of the potential for the Cr dissolution reaction and the Gibbs free energy ($\Delta G$) for the overall corrosion reaction would then become less negative ($\Delta G = - nFE_a$), meaning a decreased driving force for dealloying.

**Supplementary Table 2:** Trace impurities in pristine microwire - composition analysis. (supplementary reference [1])

Ti and W were analyzed by ICP-MS. Other trace impurity analysis by ICP-OES. The balances are Ni: 78.92 +/- 1.58% and Cr: 19.71 +/- 0.39% (by ICP-OES).

| | | Al | Ca | Cu | Fe | Mn | Si | Zr | Ti* | W* |
|---|---|---|---|---|---|---|---|---|---|---|
| **Ni-20Cr** | μg $g^{-1}$ wire | 1305 | 428 | 235 | 1957 | 232 | 11564 | 744 | <DL | 27 |
| **STDEV** | | 40 | 5 | 3 | 30 | 9 | 384 | 14 | <DL | 1 |

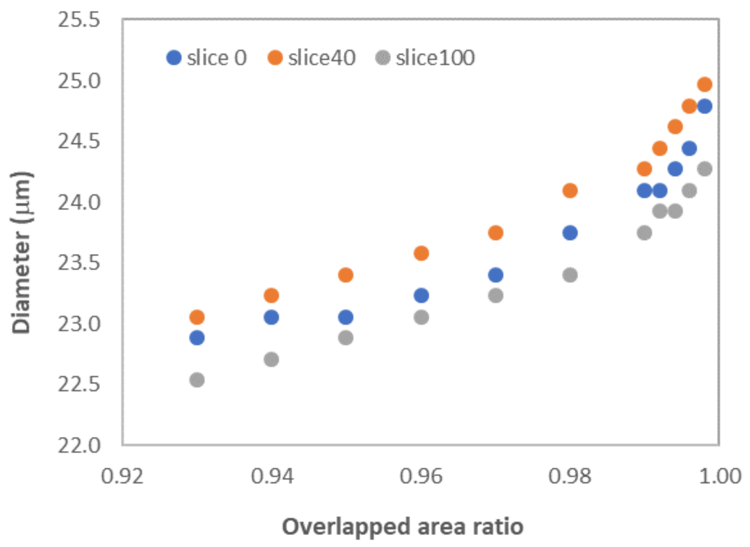


**Supplementary Figure 3:** Plot of the diameter of the fitted circle *vs.* the overlapped area ratio between the fitted circle and the solid phase (by voxel counting) with one representative scan. When the overlapped area ratio was higher than 0.98, the diameter of the fitted circle increased more because the fitted circle started to cover a small number of artifacts from the segmentation outside the solid phase region. Therefore, the ratio used for all time points was determined by calculating the intercept of the two trend lines.

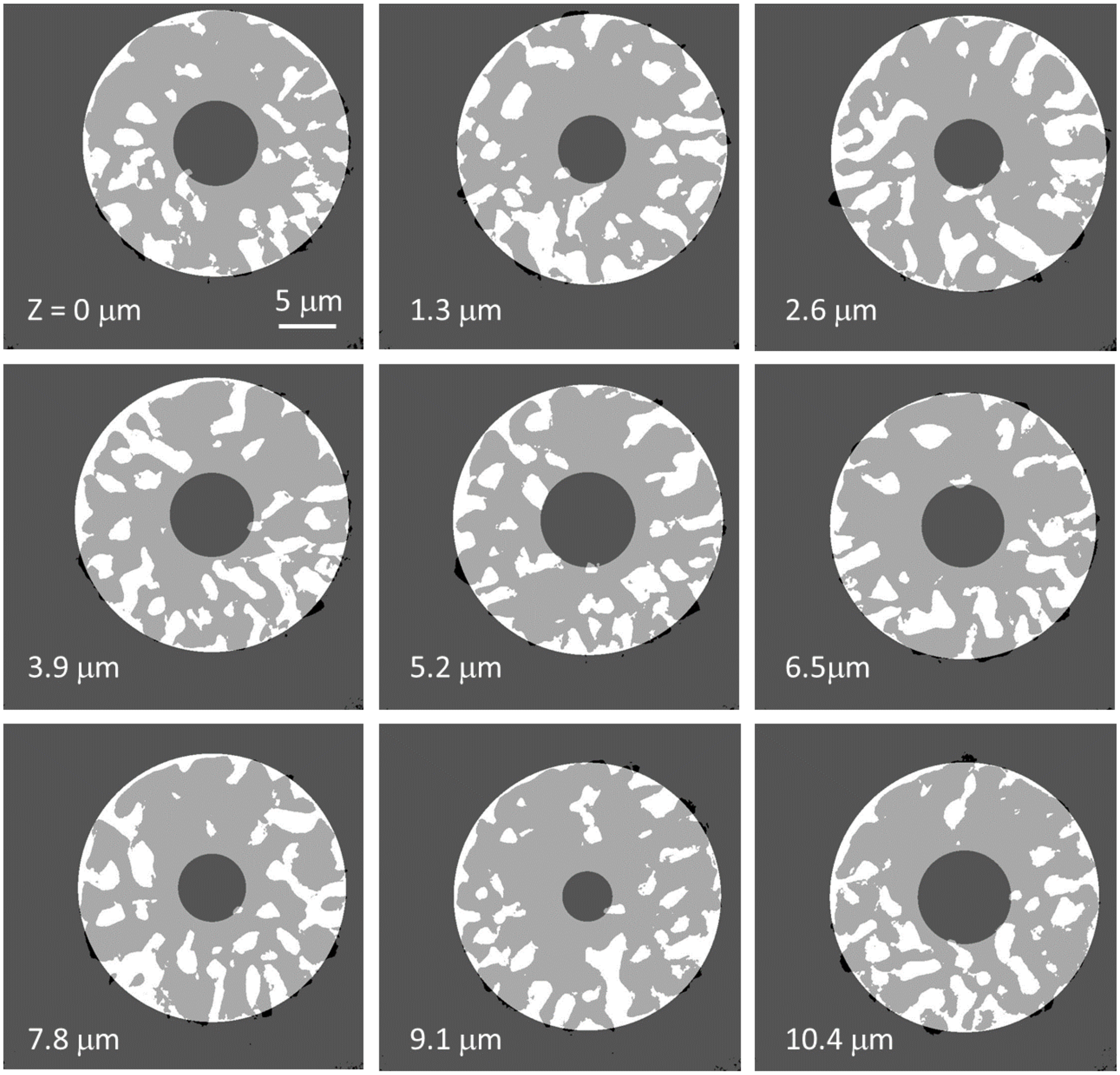


**Supplementary Figure 4:** The fitting results of inner and outer circles of segmented pseudo XY planes for a dealloying time of 16.6 minutes at different Z locations (along the wire's length). The inner circle covers at least 99.4% of the non-corroded region, while the larger circle overlaps at least 98.8% of the whole samples in XY plane. The corrosion distance was measured by the radius difference between these two fitted circles.

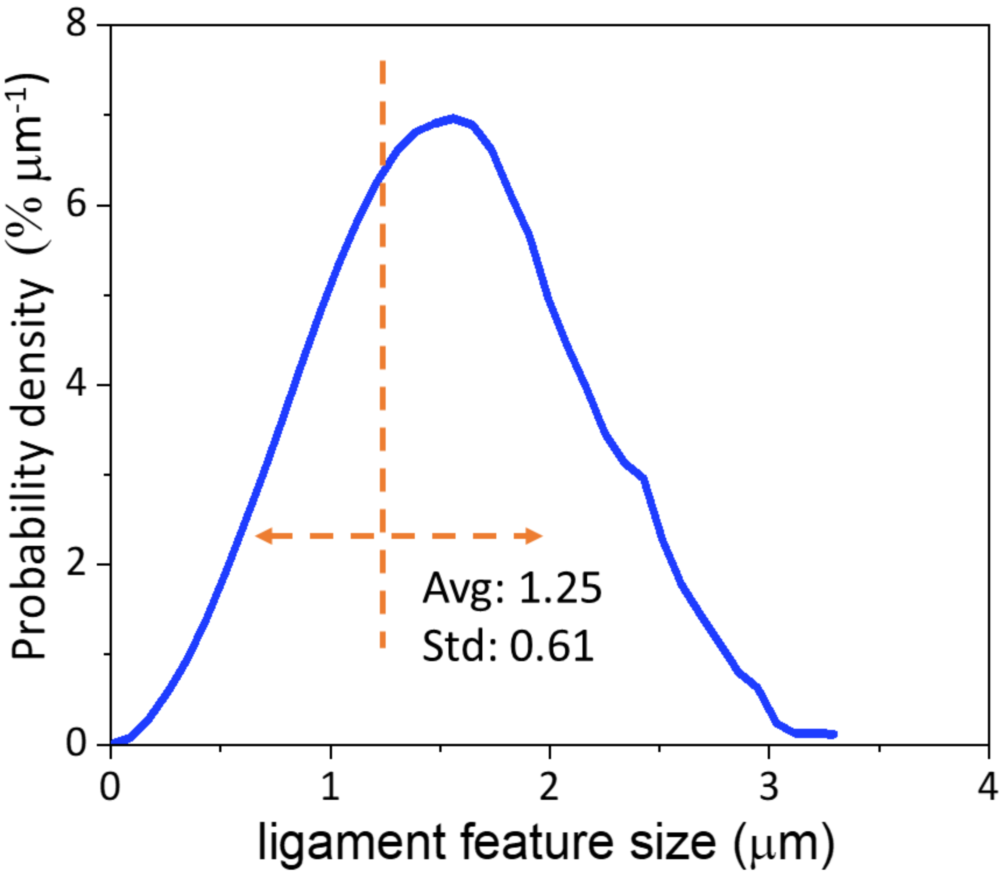


**Supplementary Figure 5:** An example of the ligament feature size distribution characterization of the bicontinuous structure, quantified for the dealloying time of 61.0 minutes. The distribution was then used to calculate the average feature size (Avg) and the standard deviation (Std), corresponding to the feature size distribution in Fig. 2**d**. The average feature size with the distribution range were for each of the reaction time point based on the 3D tomographic reconstruction, and shown in Fig 2**d**.

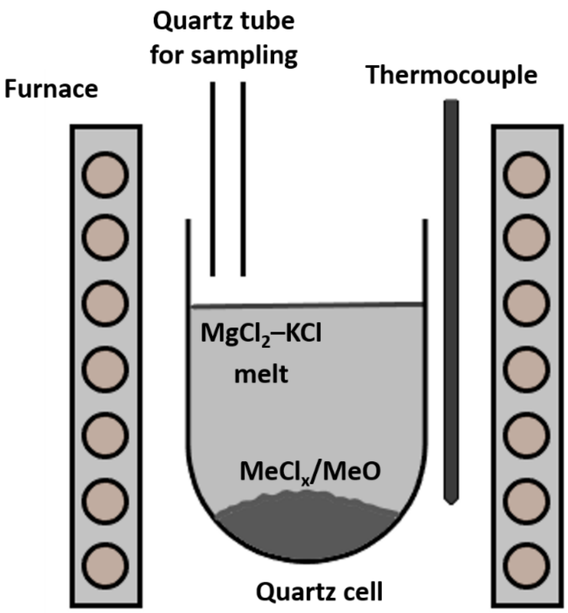


**Supplementary Figure 6:** Schematic diagram of the experimental apparatus for the solubility measurements.

Supplementary reference:

1. Ronne A*, et al.* Revealing 3D Morphological and Chemical Evolution Mechanisms of Metals in Molten Salt by Multimodal Microscopy. *Acs Applied Materials & Interfaces* **12**, 17321-17333 (2020).
