# Supplementary material for: Formation of three-dimensional bicontinuous structures via molten salt dealloying studied in real-time by in situ synchrotron X-ray nano-tomography
Source: Nat Commun. 2021 Jun 9;12:3441. doi: 10.1038/s41467-021-23598-8 (PMC8190292; doi:10.1038/s41467-021-23598-8)
Supplement: Supplementary file 8 — Description of Additional Supplementary Files [file 41467_2021_23598_MOESM8_ESM.docx]

Description of additional supplementary information

Title: Supplementary Movies

Description: Supplementary Movie 1 An in situ movie of Ni-20Cr reaction in KCl-MgCl2 at 800 ℃ from XY cross-sectional view.

Title: Supplementary Movie 2

Description: An in situ movie of Ni-20Cr reaction in KCl-MgCl2 at 800 ℃ from XZ cross-sectional view.

Title: Supplementary Movie 3

Description: An in situ movie of Ni-20Cr reaction in KCl-MgCl2 at 800 ℃ from 3D volume rendering view.

Title: Supplementary Movie 4

Description: An in situ movie of Ni-20Cr reaction in KCl-MgCl2 at 800 ℃ shows the ligament pinch-off at location 1.

Title: Supplementary Movie 5

Description: An in situ movie of Ni-20Cr reaction in KCl-MgCl2 at 800 ℃ shows the ligament pinch-off at location 2.
